# P-129. Clinical Utility and Optimisation Strategies of Rapid Molecular Testing in Central nervous system (CNS) Infections: Insights from an Indian tertiary care centre

**DOI:** 10.1093/ofid/ofaf695.356

**Published:** 2026-01-11

**Authors:** Yash Khatod, Gopal Krishana Bohra, Deepak Kumar, Neetha Ramankutty, Samhita Panda, Ravisekhar Gadepalli, Navneet Kaur, Tejasvi kanagiri

**Affiliations:** MGUMST , Jaipur, Rajasthan, India; All India Institute of Medical Sciences, Jodhpur, Jodhpur, Rajasthan, India; All India Institute of Medical Sciences, Jodhpur India, Jodhpur, Rajasthan, India; All India Institute of Medical Sciences, Jodhpur, Jodhpur, Rajasthan, India; All India Institute of Medical Sciences, Jodhpur, Jodhpur, Rajasthan, India; AIIMS, Jodhpur, Jodhpur, Rajasthan, India; All India Institute of Medical Sciences, Jodhpur, Jodhpur, Rajasthan, India; AIIMS, Jodhpur, Jodhpur, Rajasthan, India

## Abstract

**Background:**

Central nervous system (CNS) infections pose a significant health threat due to their high mortality and morbidity. Rapid molecular tests (RMTs) offer early pathogen identification and can improve outcomes in such cases. However, in resource-limited settings like India, high cost and distinct epidemiological patterns, such as a higher prevalence of CNS-tuberculosis, may hinder their widespread use. This study investigates the clinical utility and optimal-utilization strategies for multiplex PCR-based meningitis/encephalitis panels and Gene-Xpert MTB/RIF Ultra as RMTs, covering major pathogen prevalent in Indian setup and impact on clinical outcomes.

**Figure:**
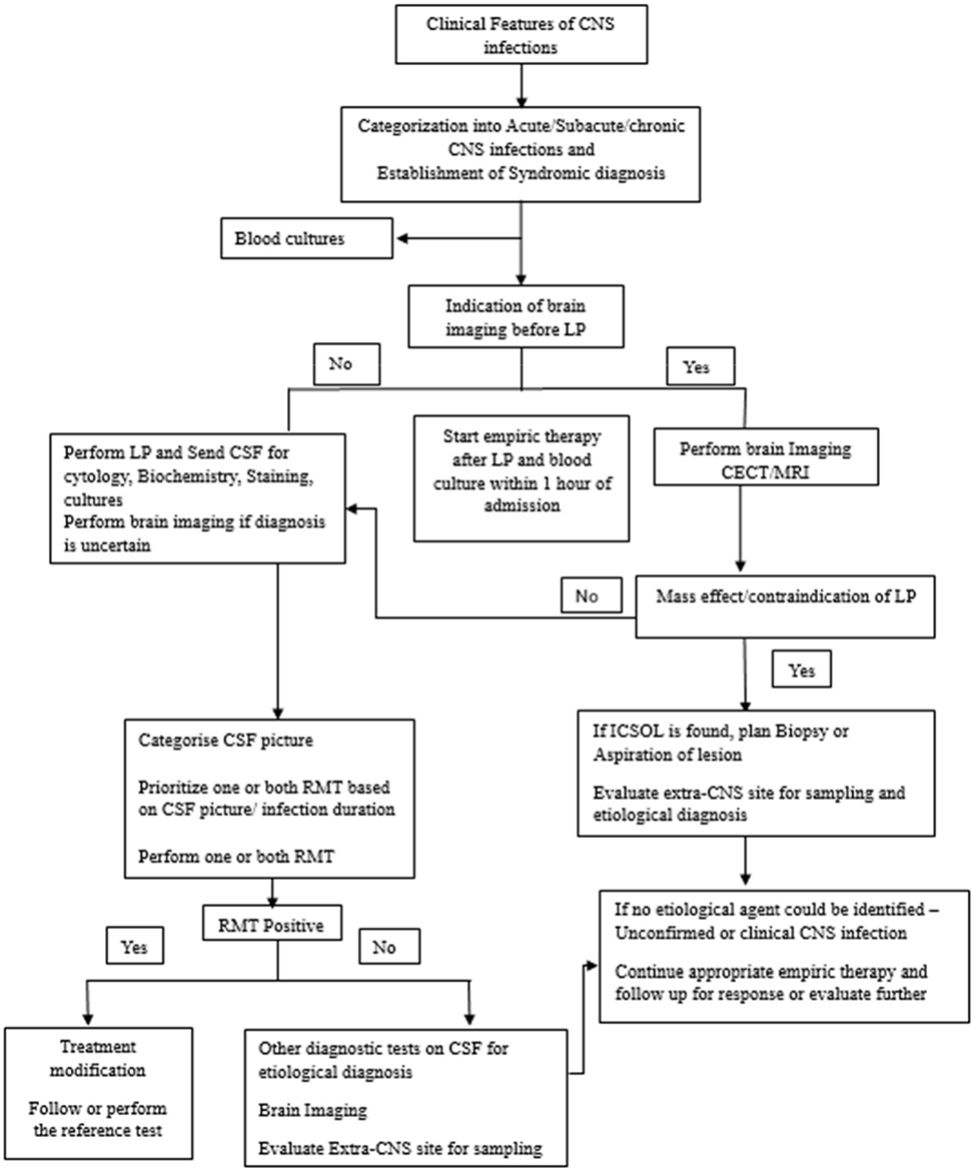
Methodology utilised for optimising RMT (Biofire ME panel or Gene Xpert ultra) by categorisation of CSF picture RMT- Rapid molecular tests; CNS- central nervous system; CSF- cerebrospinal fluid; ICSOL- Intracranial space occupying lesion

**Figure:**
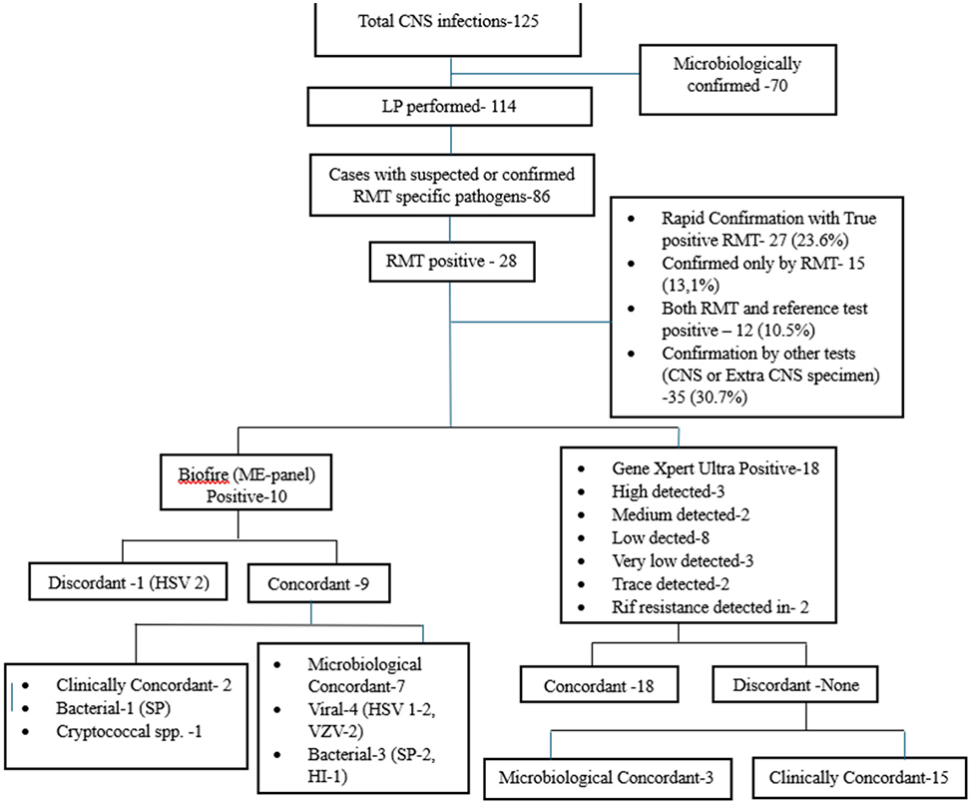
Pattern of positivity of various rapid molecular tests (RMT) on CSF and their clinical and microbiological concordance HSV- Herpes simplex virus, VZV- Varicella zoster virus, SP- Streptococcus pneumoniae, HI- Hemophilus influenzae, ME – Meningitis/Encephalitis

**Methods:**

Adult patients with community-acquired CNS infections were included, excluding those with contraindications for lumbar puncture or non-infectious aetiologies. Based on the Cerebrospinal fluid (CSF) profile, one or both Bio Fire® Meningitis/Encephalitis panel and the Gene-Xpert® MTB/RIF Ultra were performed, prioritizing either test accordingly. Diagnostic yield and clinical outcomes, including the Glasgow Outcome Scale (GOS) and duration of hospital stay, were compared between RMT-positive and RMT-negative cases.

**Figure:**
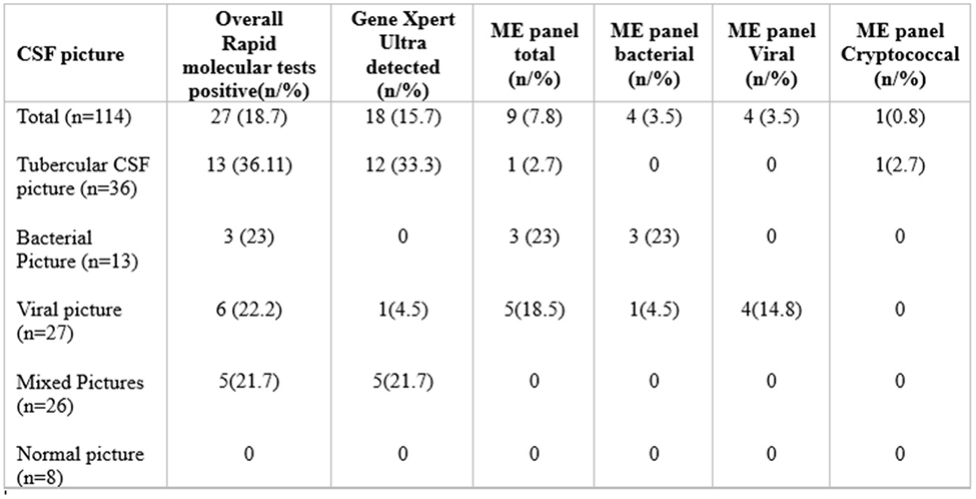
Patterns of microbiologically and/or clinically concordant positivity of the two RMT in various CSF profiles studied.

**Results:**

Out of total 114 cases, RMTs identified pathogens in 28 (24.5%) cases, with 27 (23.6%) results being clinically and/or microbiologically concordant. The CSF profile had good predictability for the outcome of RMT as only 1 sample with Tubercular and Viral profile and none with bacterial profile had deviation from expected result. The diagnostic yield of RMTs was significantly higher (31.3%) compared to reference tests (13.9%, p=0.0065). RMT positive cases had significantly better outcome, complete recovery, antibiotics downgrading, and hospital stay as compared to RMT negative cases.

**Conclusion:**

Inclusion of both Gene Xpert Ultra and ME syndromic panels in diagnostic algorithms for early and comprehensive identification of pathogens related to CNS infections is crucial for improved outcomes in developing countries like India. The CSF profile and clinical scoring system can guide in prioritising and optimising these tests.

**Disclosures:**

All Authors: No reported disclosures

